# Trajectories of Work-Related Functional Impairment prior to Suicide

**DOI:** 10.1371/journal.pone.0139937

**Published:** 2015-10-07

**Authors:** Mo Wang, Charlotte Björkenstam, Kristina Alexanderson, Bo Runeson, Petter Tinghög, Ellenor Mittendorfer-Rutz

**Affiliations:** 1 Department of Clinical Neuroscience, Division of Insurance Medicine, Karolinska Institutet, Stockholm, Sweden; 2 Department of Clinical Neuroscience, Centre for Psychiatric Research, Karolinska Institutet, Stockholm, Sweden; Medical University of Vienna, AUSTRIA

## Abstract

**Background:**

Work-related functional impairment in terms of sickness absence and disability pension (SA/DP) has been reported to be associated with subsequent suicide. However, there is only limited knowledge on SA/DP patterns prior to suicide. The aim was to identify trajectories of work-related functional impairment prior to suicide and to describe associations of socio-demographic and medical factors with such trajectories.

**Methods:**

This is a population-based retrospective cohort study of the 4 209 individuals aged 22–65 years who committed suicide during 2007–2010 in Sweden. Work-related functional impairment was measured as mean annual number of months of SA/DP. We analyzed trajectories of SA/DP during five years prior to suicide (i.e., 2002–2009) by a group-based trajectory method. Associations between socio-demographic and medical factors with different groups of trajectories were estimated by chi^2^-test and multinomial logistic regression.

**Results:**

Five different functional impairment trajectory groups were identified prior to suicide. One group had constant low levels of SA/DP (46%), while 30% had constant high levels of SA/DP. Two groups (16%) showed increasing number of SA/DP months. The remaining 7% showed decreasing number of SA/DP months before the suicide. Sex, age, educational level, family situation, and diagnosis-specific healthcare were significantly associated with different trajectory groups (Likelihood ratio X^2^ tests <0.05). A larger proportion of higher educated and younger men with a lower proportion of previous suicide attempts were found in the group with constant low levels. Opposite characteristics were displayed in the group with constant high levels.

**Conclusions:**

This study identified five different groups of work-related functional impairment trajectories before suicide. These differences might be partly explained by the variations in socio-demographic profiles and health care consumptions five years before suicide.

## Introduction

Suicide is a major public health issue, also globally with nearly one million annual suicide deaths [1]. Worldwide, suicide is among the leading causes of death in working aged individuals [1].

People of working ages are frequently recommended sickness absence (SA) in healthcare, that is, given a sick note or granted disability pension (DP) according to reduced work capacity caused by injuries or diseases [2]. Still, SA/DP are seldom considered in studies of suicide risk, despite that mental disorders, the main underlying reason for suicidal behavior, are often associated with work-related functional impairment in terms of SA/DP [2–6]. Moreover, work-related functional impairment is likely to be related with stressful life events caused by financial hardship, social isolation, and unhealthy behaviour, which could increase the risk of suicide [7, 8]. Both prevalence and duration of sick leave, in general and with specific diagnoses have been reported to be associated with suicidal behavior [9–12]. Previous studies also reported higher risks of suicidal behavior in individuals on DP [5, 6, 13–15]. Most of these studies have not been based on longitudinal data of SA/DP. Work-related functional impairment has been demonstrated to have different trajectories before diagnosing of chronic diseases, e.g. diabetes [16]. Knowledge on work-related trajectories may be beneficial for early identifying patients at higher risk. Still, to date there is no information on differences in trajectories of SA/DP prior to suicide.

Moreover, socio-demographic and health care related factors have been identified to be associated with SA and DP [17–19]. Information on these factors may facilitate the characterization of different SA/DP trajectories prior to suicide. To the best of our knowledge, there is to date no study investigating different trajectories of SA/DP and identifying socio-demographic and medical factors associated with trajectories of SA/DP among individuals with subsequent suicide. The use of such dynamic approaches in epidemiological research has been recommended for better insights in psychopathological development [20].

### Aim

The aim of this study was to identify trajectories of work-related functional impairment in terms of SA/DP prior to suicide. Another aim was to describe associations of socio-demographic and medical factors in the different trajectory groups of work-related functional impairment before suicide in order to gain a better understanding of these trajectories.

## Materials and Methods

### Study design

We conducted a population-based retrospective cohort study by means of merging different Swedish nationwide registers. We selected all the 4 209 individuals who committed suicide during 2007–2010 when aged 22–65 years, from the Swedish Cause of Death Register. Suicide is often underreported or reported as undetermined intent [21], therefore, suicide was defined according to the International Classification of Diseases (ICD) version 10 codes X60-X84 and Y10-Y34 (undetermined intent) [22, 23].

The individuals’ unique personal identity number was used to link de-identified annual data from nationwide registers from Statistics Sweden and the National Board of Health and Welfare for the five years preceding the year of suicide. We used an annual time-scale where T–5 represents 5 years prior to suicide (i.e., 2002–2005). The time during T–5 to T–1 (i.e., 2002–2009) was applied to explore how trajectories of SA/DP developed over time before suicide.

### Socio-demographic and medical characteristics

Socio-demographic characteristics included sex, age, educational level, type of place of residence, country of birth, and family situation and were obtained from Statistics Sweden. The variables were measured at T–5 and categorized as indicated in Table 1.

**Table 1:** Descriptive statistics for women (n = 1 181) and men (3 028) in Sweden, aged 22–65, who committed suicide in 2007–2010 (N = 4 209).

*Characteristics*	All		Women		Men	
	n	%	n	%	n	%
***Socio-demographic characteristics***						
Age * ^a^						
17–27	786	18.7	176	14.9	610	20.1
28–38	873	20.7	257	21.8	616	20.3
39–49	1 276	30.3	368	31.2	908	30.0
50–61	1 274	30.3	380	32.2	894	29.5
Education (years) * ^a^						
Compulsory (≤9)	1 290	30.6	321	27.2	969	32.0
High school (10–12)	2 105	50.0	570	48.3	1 535	50.7
University (>12)	778	18.5	278	23.5	500	16.5
Missing	36	0.9	12	1.0	24	0.8
Country of birth ^a^						
Sweden	3 635	86.4	1 011	85.6	2 624	86.7
Other Nordic countries	209	5.0	61	5.2	148	4.9
EU25 without Northern European countries	97	2.3	37	3.1	60	2.0
Rest of the world	268	6.4	72	6.1	196	6.5
Type of place of residence* ^a^ ^,^ ^b^						
Big cities	1 459	34.7	453	38.4	1 006	33.2
Medium sized cities	1 477	35.1	417	35.3	1 060	35.0
Small towns/villages	1 237	29.4	299	25.3	938	31.0
Missing	36	0.9	12	1.0	24	0.8
Family situation* ^a^						
Married^c^ living without children	367	8.7	128	10.8	239	7.9
Married^c^ living with children	877	20.8	233	19.7	644	21.3
Single^d^ living without children	2 452	58.3	578	48.9	1 874	61.9
Single^d^ living with children	258	6.1	185	15.7	73	2.4
Adolescents living with parents, 16–20 years	219	5.2	45	3.8	174	5.7
Missing	36	0.9	12	1.0	24	0.8
***Medical characteristics***						
Hospital stay due to mental disorders (days) * ^a^ ^,^ ^e^						
No hospital stay	3 784	89.9	1 009	85.4	2 775	91.6
1 to 11	226	5.4	87	7.4	139	4.6
>11	199	4.7	85	7.2	114	3.8
Hospital stay due to somatic disorders (days) * ^a^ ^,^ ^e^						
No hospital stay	3 663	87.0	970	82.1	2 693	88.9
1 to 3	308	7.3	110	9.3	198	6.5
>3	238	5.7	101	8.6	137	4.5
Outpatient care visits due to mental disorders (visits) * ^a^ ^,^ ^f^						
No visits	3 722	88.4	994	84.2	2 728	90.1
1 to 2	305	7.2	111	9.4	194	6.4
>2	182	4.3	76	6.4	106	3.5
Outpatient care visits due to somatic disorders (visits) * ^a^ ^,^ ^f^						
No visits	2 243	53.3	499	42.3	1 744	57.6
1 to 2	1 010	24.0	297	25.1	713	23.5
>2	956	22.7	385	32.6	571	18.9
Suicide attempt (inpatient care) * ^a^						
No suicide attempt	4 109	97.6	1 127	95.4	2 982	98.5
Suicide attempt	100	2.4	54	4.6	46	1.5

* Significant sex differences.

^a^ Measured at T–5.

^b^ Place of residence: big cities: Stockholm, Gothenburg and Malmö; medium sized cities: cities with more than 90 000 inhabitants within 30 km distance from the centre of the city; small cities/villages.

^c^ Married includes all living with partner; cohabitant.

^d^ Single includes divorced, separated or widowed

^e^ Hospital stay due to mental/somatic disorders (days): categorized based on median length among those hospitalized.

^f^ Outpatient specialized health care visits due to mental/somatic disorders: categorized based on median visits among those with such visits.

Information on both mental and somatic diagnoses as well as suicide attempts was obtained from the National Patient Register. In-patient care was categorized according to the median length of inpatient care among those hospitalized (no inpatient care; ≤median length; >median length). A similar approach was used for outpatient specialized health care visits (no visits; ≤median visits; >median visits). Health care information was measured at T–5, as indicated in Table 1. The median number of days of inpatient care due to mental and somatic disorders was 11 and 3, respectively, among those with such care. The median number of visits to outpatient specialized health care was 2 among those with such care, both for mental and somatic disorders. Inpatient care for suicide attempt was measured at T–5 by combining ICD–10 codes X60-X84 (determined intent) and Y10-Y34 (undetermined intent) and was categorized as a dichotomous variable.

### Sickness absence and disability pension

During the study period, all people in Sweden above the age of 16 who had reduced work capacity due to disease or injury were eligible for sickness benefits if having an income from work or unemployment or parental benefits. Sickness benefit amounts to 80% of the lost income up to a certain level. Employers provided sick pay for the first 2 weeks of a sick-leave spell, thereafter employees received sickness benefits from the Social Insurance Agency (SIA). Unemployed individuals could be granted benefits from SIA from the second day of a sick-leave spell (the first day being a qualifying day for both employed and unemployed individuals) whereas self-employed individuals received sick pay from SIA according to which insurance coverage they had chosen. DP could be granted all individuals living in Sweden whose work capacity had been reduced permanently due to disease or injury. People below the age of 30 could be granted temporary disability pension if their work capacity (including capacity to study) was reduced due to disease or injury for at least one year [24]. Information on SA and DP was obtained from Statistics Sweden. We measured work-related functional impairment by combining SA and DP days and calculated mean annual number of SA/DP net days from T–5 to T–1. Because SA and DP can be for part time, we used the number of net days, where e.g., two days of half-time sickness benefits equals one net day. We then transformed the number of net days to number of months with SA/DP.

### Statistical analyses

Group-based trajectory modelling was used to estimate trajectories of SA/DP from T–5 to T–1 before suicide. This procedure is based on a mixture model that provides the capacity to identify subgroups of individuals who followed distinct trajectories during the time of observation and estimates a regression model for each discrete group [25]. First, we used the Bayesian information criterion (BIC) to test the best-fitted model related to number of groups. The BIC scores are negative values in which those closer to 0 indicate a better fit. The optimal number of groups was defined as when the BIC score did not improve any longer.

Second, we estimated associations of socio-demographic and medical characteristics in each SA/DP trajectory group by chi^2^-test and multinomial logistic regression. Possible sex differences in the proportion of characteristics in relation to different socio-demographic and medical characteristics were tested by chi^2^-test. For the chi^2^-test in relation to SA/DP trajectory groups we excluded missing values (n = 36) on “education”, “type of place of residence” and “family situation”. For the multinomial logistic regression, individuals (n = 36) with missing values were excluded from the study population. The likelihood ratio chi^2^-tests were used to evaluate whether socio-demographic and medical factors were associated with type of trajectory in the full model and Nagelkerke R^2^ were used to evaluate the strength of these associations. By consecutively excluding each factor from the full model, we calculated differences in R^2^ for each factor in order to examine the contribution of a given factor to the full model. Data processing was performed using statistical software SAS for Windows version 9.4 (SAS-based procedure “Traj”) and SPSS for Windows version 22.0 (chi^2^-test and multinomial logistic regression).

### Ethics approval

The study population was based on linkage of several public national registers. Ethical vetting is always required when using register data in Sweden. The ethical vetting is performed by regional ethical review boards and the risk appraisal associated with the Law on Public Disclosure and Secrecy is done by data owners. The ethical review boards can however waive the requirement to consult the data subjects (or in case of minors/children the next of kin, careers or guardians) directly to obtain their informed consent, and will often do so if the research is supported by the ethical review board and the data has already been collected in some other context. Also, the institutional review board/ethics committee waived the need for written informed consent from the participants. Patient records/information was anonymized and de-identified prior to analysis by the authority, Statistics Sweden, which was responsible for data linkage. Researchers received de-identified data. According to these standards in Sweden this project has been evaluated and approved by the Regional Ethical Review Board of Karolinska Institutet, Stockholm, Sweden.

## Results

In Table 1 descriptive statistics of the cohort with regard to characteristics on socio-demographics and medical factors is shown for all as well as stratified by sex. Of the 4 209 individuals who committed suicide during 2007–2010, 1 181 (29.1%) were women. Five years before the suicide (T–5), the majority of the cohort was of middle or older age (39–61 years, 60.6%), had achieved an education at high school level (50.0%), was born in Sweden (86.4%), and was single and living without children at home (58.3%). Regarding in- or outpatient care at T–5, the majority had had neither mental (89.9% vs. 88.4%) nor somatic (87.0% vs. 53.3%) health care. The overwhelming proportion (97.6%) had not attempted suicide at T–5. There were significant sex differences regarding all socio-demographic and medical factors (P<0.05) except for “country of birth”. Notably, more men than women were single without children (61.9% vs. 48.9%) while more women than men were single with children (15.7% vs. 2.4%).

Among those who had had inpatient care, more women than men had inpatient mental health care (14.6% vs. 8.4%) and inpatient somatic health care (17.9% compared to 11.0% among men), respectively. Also, a higher rate of the women than men had outpatient mental health care (15.8% vs. 9.9%) and outpatient somatic health care (57.7% vs. 42.4%). Moreover, at T–5 a higher rate of the women (4.6%) than men (1.5%) had had inpatient care due to attempted suicide (Table 1).

### Trajectory analyses

**Fig 1:**
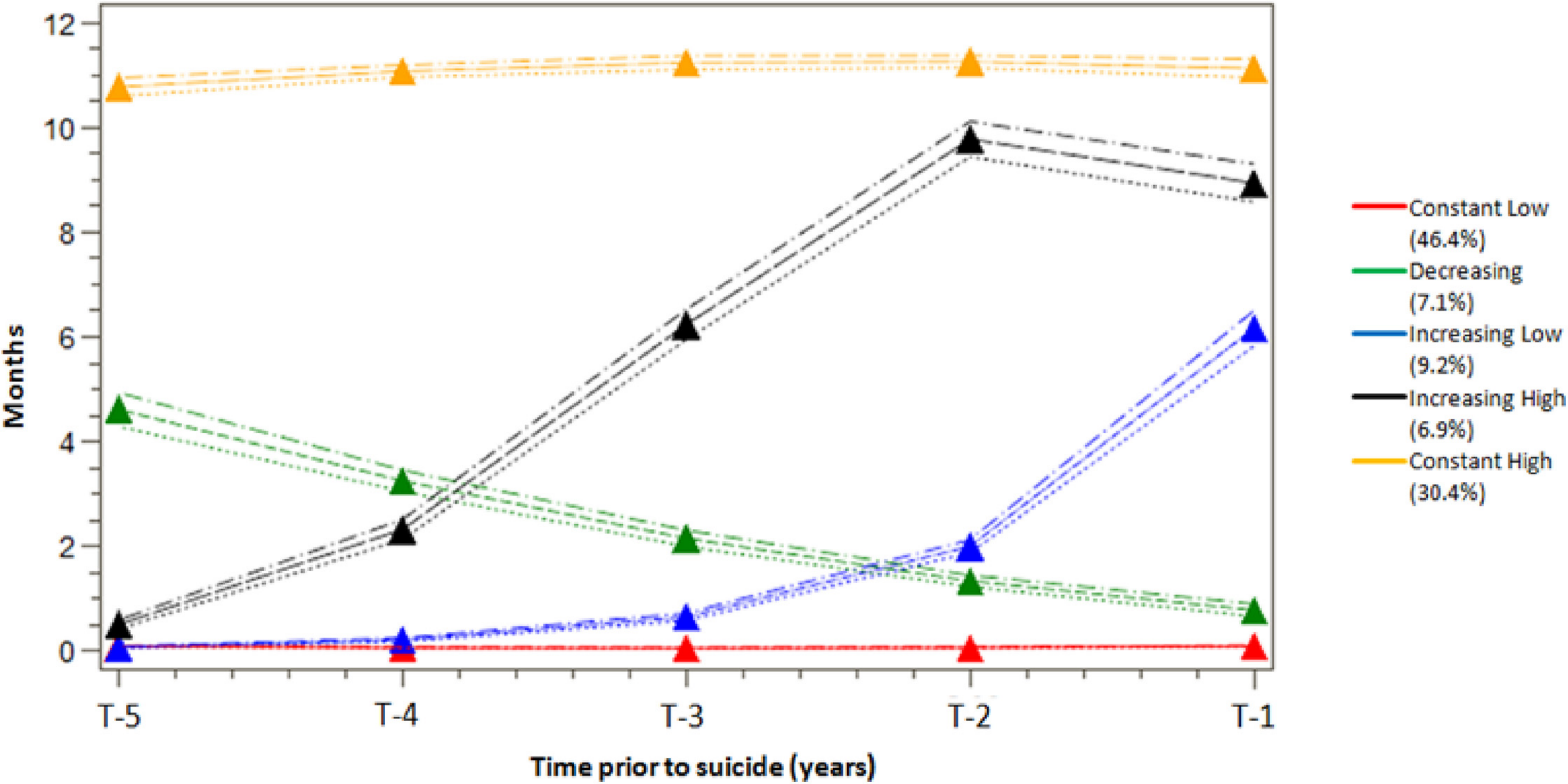
SA/DP trajectories during the five years (T–5 to T–1) prior to suicide, mean number of net SA/DP months per year. The figure displayed five different trajectories of sickness absence and disability pension (SA/DP) five years before suicide. The “Constant Low” group (red line) included individuals (46.4%) who had few month of SA/DP over the year before suicide. The “Constant high” group (yellow line) included 30.4% of the individuals with high level of SA/DP over the years. There were 9.2% of individuals belonged to the “Increasing Low” group (blue line) and 6.9% of individuals belonged to the “Increasing High” group (black line). This two groups revealed increasing of SA/DP with different speeds. The “Decreasing” group (green line) contained 7.1% of the individuals with a decrease in SA/DP over time.

In the trajectory analyses, the BIC-based procedure identified five groups of different trajectories of SA/DP months as the best fitting model (Fig 1). We labelled the five groups as follows: “Constant Low”, “Constant High”, “Increasing Low”, “Increasing High”, and “Decreasing”.

A large proportion of the individuals (46.4%) belonged to the “Constant Low” group. They constantly had few months of SA/DP over the years prior to suicide. The “Constant High” group included 30.4% of the individuals. On average, they had more than 10 months of SA/DP/year annually. There were 9.2% and 6.9% of the individuals in the “Increasing Low” and the “Increasing High” groups, respectively. Both of the groups mainly had increasing SA/DP months during the time prior to suicide with different increasing patterns. The “Increasing Low” group had less SA/DP months at T–5 and increased only marginally up until T–2, when the increase was sharp. On the other hand, the “Increasing High” group started with a strong increase of SA/DP months and continued with the strong increasing until T–2. The “Decreasing” group contained 7.1% of the individuals and showed a decrease in SA/DP over time (Fig 1).

Table 2 shows distributions and associations of socio-demographic and medical characteristics in each trajectory group. All socio-demographic and medical factors were significantly associated with the trajectory groups (P<0.05) with the exception of “country of birth” and “type of place of residence”. After controlling for all socio-demographic and medical factors in the full model, the significant results persisted. The full model explained approximately 34% (Nagelknerk R^2^) of the variance between the trajectory groups. The differences in R^2^ indicated that the different separate factors did not independently have strong effects in the full model, except for age (Diff. in R^2^ = 0.07), which had a more important role than other factors in the full model.

**Table 2:** Distributions and associations of socio-demographic and medical characteristics in each trajectory group of mean SA/DP month/year in individuals committing suicide in 2007–2010 (N = 4 209).

*Characteristics*	Constant Low	Decreasing	Increasing Low	Increasing High	Constant High	X^2^ (p-value)	Log-likelihood testX^2^(p-value)	Diff. in R^2^ *
	n (%)	n (%)	n (%)	n (%)	n (%)			
***Socio-demographic characteristics***								
Sex ^a^								
Women	356 (30.1)	101 (8.6)	117 (9.9)	94 (8.0)	513 (43.4)	195.8 (<0.01)	118.4 (<0.01)	0.02
Men	1 605 (53.0)	200 (6.6)	261 (8.6)	195 (6.4)	767 (25.3)			
Age ^a^								
17–27	532 (67.7)	37 (4.7)	101 (12.8)	53 (6.7)	63 (8.0)	399.3 (<0.01)	394.6 (<0.01)	0.07
28–38	455 (52.1)	73 (8.4)	79 (9.9)	56 (6.4)	210 (24.1)			
39–49	527 (41.3)	117 (9.2)	100 (7.8)	99 (7.8)	433 (33.9)			
50–61	447 (35.1)	74 (5.8)	98 (7.7)	81 (6.4)	574 (45.1)			
Education (years) ^a^								
Compulsory (≤9)	587 (45.5)	80 (6.2)	103 (8.0)	91 (7.1)	429 (33.3)	25.6 (<0.01)	40.2 (<0.01)	0.01
High school (10–12)	936 (44.5)	172 (8.2)	198 (9.4)	145 (6.9)	654 (31.1)			
University (>12)	407 (52.3)	49 (6.3)	74 (9.5)	51(6.6)	197 (25.3)			
Country of birth ^a^								
Sweden	1 685 (46.4)	264 (7.3)	335 (9.2)	247 (6.8)	1 104 (30.4)	14.2 (0.29)	4.7 (0.97)	<0.01
Other Nordic countries	90 (43.1)	13 (6.2)	12 (5.7)	14 (6.7)	80 (38.3)			
EU25 without Northern European countries	46 (47.4)	7 (7.2)	6 (6.2)	8 (8.2)	30 (30.9)			
Rest of the world	140 (52.2)	17 (6.3)	25 (9.3)	20 (7.5)	66 (24.6)			
Type of place of residence ^a^ ^,^ ^b^								
Big cities	659 (45.2)	108 (7.4)	141 (9.7)	102 (7.0)	449 (30.8)	5.3 (0.72)	11.9 (0.16)	<0.01
Medium sized cities	680 (46.0)	103 (7.0)	139 (9.4)	106 (7.2)	449 (30.4)			
Small towns/villages	591 (47.8)	90 (7.3)	95 (7.7)	79 (6.4)	382 (30.9)			
Family situation ^a^								
Married^c^ living without children	148 (40.3)	19 (5.2)	29 (7.9)	22 (6.0)	149 (40.6)	240.5 (<0.01)	116.8 (<0.01)	0.02
Married^c^ living with children	482 (55.0)	78 (8.9)	94 (10.7)	66 (7.5)	157 (17.9)			
Single^d^ living without children	1 050 (42.8)	172 (7.0)	200 (8.2)	169 (6.9)	861 (35.1)			
Single^d^ living with children	83 (32.2)	28 (10.9)	25 (9.7)	176 (6.6)	105 (40.7)			
Adolescents living with parents, 16–20 years	167 (76.3)	4 (1.8)	27 (12.3)	13 (5.9)	8 (3.7)			
***Medical characteristics***								
Hospital stay due to mental disorders (days) ^a^ ^,^ ^e^								
No hospital stay	1 899 (50.2)	252 (6.7)	353 (9.3)	261 (6.9)	1 019 (26.9)	277.1 (<0.01)	47.7 (<0.01)	0.01
1 to 11	38 (16.8)	22 (9.7)	18 (8.0)	15 (6.6)	133 (58.8)			
>11	24 (12.1)	27 (13.6)	7 (3.5)	13 (6.5)	128 (64.3)			
Hospital stay due to somatic disorders (days) ^a^ ^,^ ^e^								
No hospital stay	1 835 (50.1)	246 (6.7)	345 (9.4)	247 (6.7)	990 (27.0)	215.2 (<0.01)	17.3 (<0.05)	<0.01
1 to 3	89 (28.9)	29 (9.4)	23 (7.5)	26 (8.4)	141 (45.8)			
> 3	37 (15.5)	26 (10.9)	10 (4.2)	16 (6.7)	149 (62.6)			
Outpatient care visits due to mental disorders (visits) ^a^ ^,^ ^f^								
No visits	1 875 (50.4)	256 (6.9)	346 (9.3)	247 (6.6)	998 (26.8)	257.5 (<0.01)	115.7 (<0.01)	0.02
1 to 2	69 (22.6)	22 (7.2)	22 (7.2)	25 (8.2)	167 (54.8)			
>2	17 (9.3)	23 (12.6)	10 (5.5)	17 (9.3)	115 (63.2)			
Outpatient care visits due to somatic disorders (visits) ^a^ ^,^ ^f^								
No visits	1 319 (58.8)	124 (5.5)	217 (9.7)	142 (6.3)	441 (19.7)	538.5 (<0.01)	229.2 (<0.01)	0.04
1 to 2	442 (43.8)	76 (7.5)	109 (10.8)	76 (7.5)	307 (30.4)			
> 2	200 (20.9)	101 (10.6)	52 (5.4)	71 (7.4)	532 (55.6)			
Suicide attempt (inpatient care) ^a^								
No suicide attempt	1 954 (47.6)	290 (7.1)	370 (9.0)	282 (6.9)	1 213 (29.5)	81.8 (<0.01)	12.8 (<0.05)	<0.01
Suicide attempt	7 (7.0)	11 (11.0)	8 (8.0)	7 (7.0)	67 (67.0)			

* Difference in Nagelkerke R^2^ between model including tested variable and model without tested variable. Nagelkerke R^2^ for full model is 0.34.

^a^ Measured at T–5.

^b^ Type of place of residence: big cities: Stockholm, Gothenburg and Malmö; medium sized cities: cities with more than 90 000 inhabitants within 30 km distance from the centre of the city; small cities/villages.

^c^ Married includes all living with partner; cohabitant.

^d^ Single includes divorced, separated or widowed

^e^ Hospital stay due to mental/somatic disorders (days): categorized based on median length among those hospitalized.

^f^ Outpatient specialized health care visits due to mental/somatic disorders: categorized based on median visits among those with such visits.

In Table 2, it is shown that younger men and individuals with higher education, who were adolescents living with their parents, as well as those who had neither mental nor somatic inpatient care nor suicide attempt were overrepresented in the “Constant Low” group. Still, 43.8% of the individuals in this group had had outpatient somatic health care with 1–2 visits. In contrast, these factors were underrepresented in “Constant High” group. Instead, this group predominantly comprised older women and individuals with lower education and those with more health care and suicide attempts.

The “Decreasing” group consisted mainly of individuals of female sex, middle age (39–49 years), with high school education, and who were living single with children. Regarding medical characteristics, around 10–15% had attempted suicide or had mental or somatic in- or outpatient care exceeding the median number of days or visits.

The two groups with increasing SA/DP months showed slightly different distributions of socio-demographic and medical characteristics. The “Increasing Low” group tended to be younger, with higher education and with less somatic and mental inpatient and mental outpatient health care than the “Increasing High” group.

## Discussions

In this study of 4 209 people of working age in Sweden who committed suicide 2007–2010, we found five different groups of trajectories of work-related functional impairment, measured as previous number of sickness absence and disability pension (SA/DP) months over the five years preceding suicide. Nearly half of the suicide victims had few annual months of SA/DP while one third belonged to a group with more than 10 months yearly of SA/DP. There were also two smaller groups (16.1%) with increasing trends of SA/DP months preceding suicide, and one group with decreasing number of months (7% of the individuals). Sex, age, education, family situation, and diagnosis-specific health care were significantly associated with different trajectory groups.

### Methodological considerations

To the best of our knowledge, this is the first study investigating trajectories of work-related functional impairment in terms of SA/DP prior to suicide. The population-based cohort design, including all individuals aged 22–65 years in the entire country of Sweden, offered satisfactory statistical power for the analyses of the trajectories of SA/DP months prior to suicide. Another strength is that we used data on suicide, SA/DP, socio-demographic, and medical factors from nationwide registers that are of good quality [26, 27]. Still, underreported suicide may occur.

Some limitations of the study and considerations when interpreting our findings are important to mention. Information on sick-leave spells <14 days among employed individuals was not available. This means that for employed individuals the number of SA days contributing to the combined number of SA/DP days might be an underestimation. We only included suicide attempts that required inpatient care, that is, the medically most serious ones. This means that suicide attempters who had outpatient care or did not seek health care were not included. Further, there might be other socio-demographic or medical factors associated with trajectories of SA/DP than those we have studied.

### Trajectories of work-related functional impairment

Our findings with regard to five different trajectories of SA/DP prior to suicide reflect the general knowledge that suicide victims comprise a heterogeneous group with regard to etiology, health care seeking behaviour, the suicidal process, and underlying diseases [28]. We found that a large proportion of the individuals either showed stable high levels (30.4%) or increasing number (16.1%) of annual SA/DP months preceding suicide. This is in contrast to much lower levels or lower proportions of high/increasing work disability in individuals prior to multiple sclerosis and first diagnosis of diabetes in recent studies based on a Swedish cohort (<23%) [29], and a Finnish (<29%) and a French (<13%) occupational cohort [16]. High levels of SA/DP seem to be associated with the type and severity of the underlying diagnoses of SA/DP [30, 31]. In Western countries, up to 95% of suicide victims suffered from a mental disorder [28]. Also, potential social isolation and unhealthy life styles associated with long SA/DP might have an effect on the aggravation of symptoms, which might increase the risk of suicide [7]. On the other hand, approximately half of the individuals (46.4%) had few SA/DP months before suicide. This compares to much higher proportions of individuals with low levels of work disability prior to multiple sclerosis and first diagnosis of diabetes in recent studies based on a Sweden cohort (>50%) (29), and a Finnish (>70%) and a French (>80%) occupational cohort [16].

Our results indicate that different SA/DP trajectories can be described by using information on socio-demographics and health care consumptions. Age showed the strongest associations in the full model. According to previous research, SA/DP rates increase with age [28, 32, 33]. It is thus, reasonable to find older individuals in the “Constant High” group with many months of SA/DP whereas higher rates of younger people were included in the “Constant Low” group with few SA/DP months at T–5.

Also, health care consumption was associated with how SA/DP developed over time prior to suicide. Long mental and somatic hospital stays and more frequent out-patient visits at T–5, as measures of type and severity of the underlying disorders, were found both in the “Constant High” and “Increasing High” group. The “Decreasing” group also had high level of health care consumptions at T–5. The decreasing SA/DP months might reflect improvement of their health conditions or inadequate treatment or rehabilitation.

On the other hand, the “Constant Low” and the “Increasing Low” groups included a larger proportion of individuals with less health care consumption at T–5. Regarding the “Increasing Low” group, the few months of SA/DP at T–5 could be interpreted by the better health condition in terms of less in- or outpatient health care. The increasing number of average months with SA/DP might reflect deterioration of somatic or mental health. One possible explanation of the lower health care consumption in the “Constant Low” group is that the group also comprised a larger proportion of young men. Young men are sometimes reported to have a higher threshold for reporting health complaints and are less help-seeking than their female counterparts [34, 35]. Young males also have less frequent previous suicide attempts than females when they commit suicide [36]. Still, 43.8% of the individuals in the "Constant Low" group had outpatient visits due to somatic health care at T–5. Individuals who had contact with somatic health care may have undiagnosed or untreated depression, which has been shown to be associated with subsequent suicide [37, 38].

## Conclusions

This study showed different previous trajectories of work-related functional impairment among individuals with subsequent suicide. While nearly half of the suicide victims had low levels of work-related functional impairment in the five preceding years, around a quarter and a third had decreasing/increasing and constantly high levels, respectively. Socio-demographic and medical factors were associated with different SA/DP trajectories.
